# Smart nanoplatform for sequential drug release and enhanced chemo-thermal effect of dual drug loaded gold nanorod vesicles for cancer therapy

**DOI:** 10.1186/s12951-019-0473-3

**Published:** 2019-03-27

**Authors:** Falian Zhu, Guozhu Tan, Yingtao Zhong, Yaodong Jiang, Lulu Cai, Zhiqiang Yu, Shuwen Liu, Fei Ren

**Affiliations:** 1 Department of Pharmacy, Nanfang Hospital, School of Pharmaceutical Sciences, Guangdong Provincial Key Laboratory of New Drug Screening, Southern Medical University, Guangzhou, 510515 China; 2 Department of Urology, Nanfang Hospital, Southern Medical University, Guangzhou, 510515 China; 3 Personalized Drug Therapy Key Laboratory of Sichuan Province, Sichuan Academy of Medical Sciences and Sichuan Provincial People’s Hospital, School of Medicine, University of Electronic Science and Technology of China, Chengdu, 610072 Sichuan China; 4 School of Pharmaceutical Sciences, Guangdong Provincial Key Laboratory of New Drug Screening, Southern Medical University, Guangzhou, 510515 China

**Keywords:** Stimuli-responsive, Sequential drug release, Synergistic effect, Dual drug, Combination therapy, NIR photothermal effect

## Abstract

**Background:**

The combination of multiple chemotherapeutics has been used in the clinic for enhanced cancer chemotherapy, however, frequent relapse, chemo-resistance and side effects remains therapeutic hurdles. Thus, the development of co-delivery system with enhanced targeting and synergistic different modal treatments has been proposed as promising strategies for intensive improvement of the therapeutic outcomes.

**Results:**

We fabricated a nanocarrier based on gold nanorods (Au NRs), cRGD peptide-modified and multi-stimuli-responsive paclitaxel (PTX) and curcumin (CUR) release for synergistic anticancer effect and chemo-photothermal therapy (PTX/CUR/Au NRs@cRGD). The specific banding of cRGD to *αvβ3* integrin receptor on the tumor cell surfaces facilitated the endocytosis of PTX/CUR/Au NRs@cRGD, and the near-infrared ray (NIR) further enhanced the drug release and chemotherapeutical efficiency. Compared to single drug, single model treatment or undecorated-PTX/CUR/Au NRs, the PTX/CUR/Au NRs@cRGD with a mild NIR showed significantly enhanced apoptosis and S phase arrest in three cancer cell lines in vitro, and improved drug accumulation in tumor sites as well as tumor growth inhibition in vivo.

**Conclusions:**

The tumor targeted chemo-photothermal therapy with the synergistic effect of dual drugs provided a versatile strategy for precise cancer therapy.

**Electronic supplementary material:**

The online version of this article (10.1186/s12951-019-0473-3) contains supplementary material, which is available to authorized users.

## Background

Stimuli-responsive nanocarriers have been widely investigated as potential controllable drug-release and enhanced cancer-targeting drug delivery system (DDSs) in recent years [1–3]. There are several specific materials, chemical bonds or physical interactions on/in the nanocarriers which can respond to inherent biological factors (e.g., enzymes and pH) [4, 5] as well as external signals (e.g., temperature and light) [6, 7]. As one of the ideal external stimuli, illumination possesses minimally invasive and tunable characters that can be easily achieved by adjusting the irradiation intensity. In this regard, nanocarriers with intensive absorptions in the near-infrared (NIR) region (650–900 nm) are often designed for in vivo NIR-responsive drug delivery [8–10]. In addition, NIR light can facilitate plasmonic photothermal therapy (PPTT) from the utilization of photo-absorbing agents such as gold nanorods (Au NRs), hollow gold nanospheres and gold nanocages [11–16], thereby causing tumor ablation. Unfortunately, because of the unavoidable depth-dependent decay of light scattering and absorption, the main challenge of the PPTT is the limited tissue penetration, which results in the incomplete tumor eradication in deep tissues [17, 18]. Therefore, it is necessary to develop the novel synergistic or combined therapies with PPPT other than it alone.

The combination of chemotherapy and PPTT supply feasibility to enhance the efficiency of drug delivery and promote intracellular uptake thanks to PPTT induced changes of cell membrane permeability [19, 20]. Besides, the design system of combined PPTT with chemotherapy that acquires stimuli-responsive, ultimately resulting in tumor ablation and providing more opportunities for on-demand therapy [21]. To this end, a variety of Au NRs systems have been proposed for PPTT and carrying anticancer drugs, selectively released their payloads at the controlled NIR area, resulting in synergistic chemo and photothermo-therapeutic efficacy [22–24].

Multiple chemotherapeutics combination is commonly used in clinic to improve the therapeutic activity and mitigate the side effects. The mutations of cancer cells could be reduced and the multidrug resistance (MDR) could be delayed via the combination of dual drugs which target the different signaling pathways or molecular sites [25, 26]. Paclitaxel (PTX), one of the most vital anticancer compounds, can stabilize microtubules, arrest the cancer cell cycle at the G2/M phase and induce apoptosis [27, 28]. Additionally, curcumin (CUR) is known to have broad pharmacological functions, including anticancer and anti-inflammatory functions, without any major side effects [29, 30]. Recent studies suggest that a simultaneous treatment of paclitaxel and curcumin induces down-regulation of the PI3K/Akt and nuclear factor-κB (NF-κB) signaling pathways, which lead to DNA condensation and fragmentation, as well as intracellular ROS increasing, so that results in enhanced tumor cell apoptosis, reduced the toxicity of drugs compared to using CUR or PTX alone [31, 32]. Due to the intensely lipophilic characters of PTX and CUR, many types of DDSs have been fabricated and sequentially administered to improve their bioavailability. Although there are a few nano-DDSs reported to improve the water solubility of PTX and CUR and overcome undesired side effects, their application in clinical cancer are inadequate due to single modal treatments and similar molecular sites [33–35]. Thus, all-in-one system with multiple chemotherapeutics and PPTT character for drug delivery in a controlled and triggered manner and combination of different modal treatments have been of prominent significance to realize synergistic eradication of cancer.

**Fig. 1:**
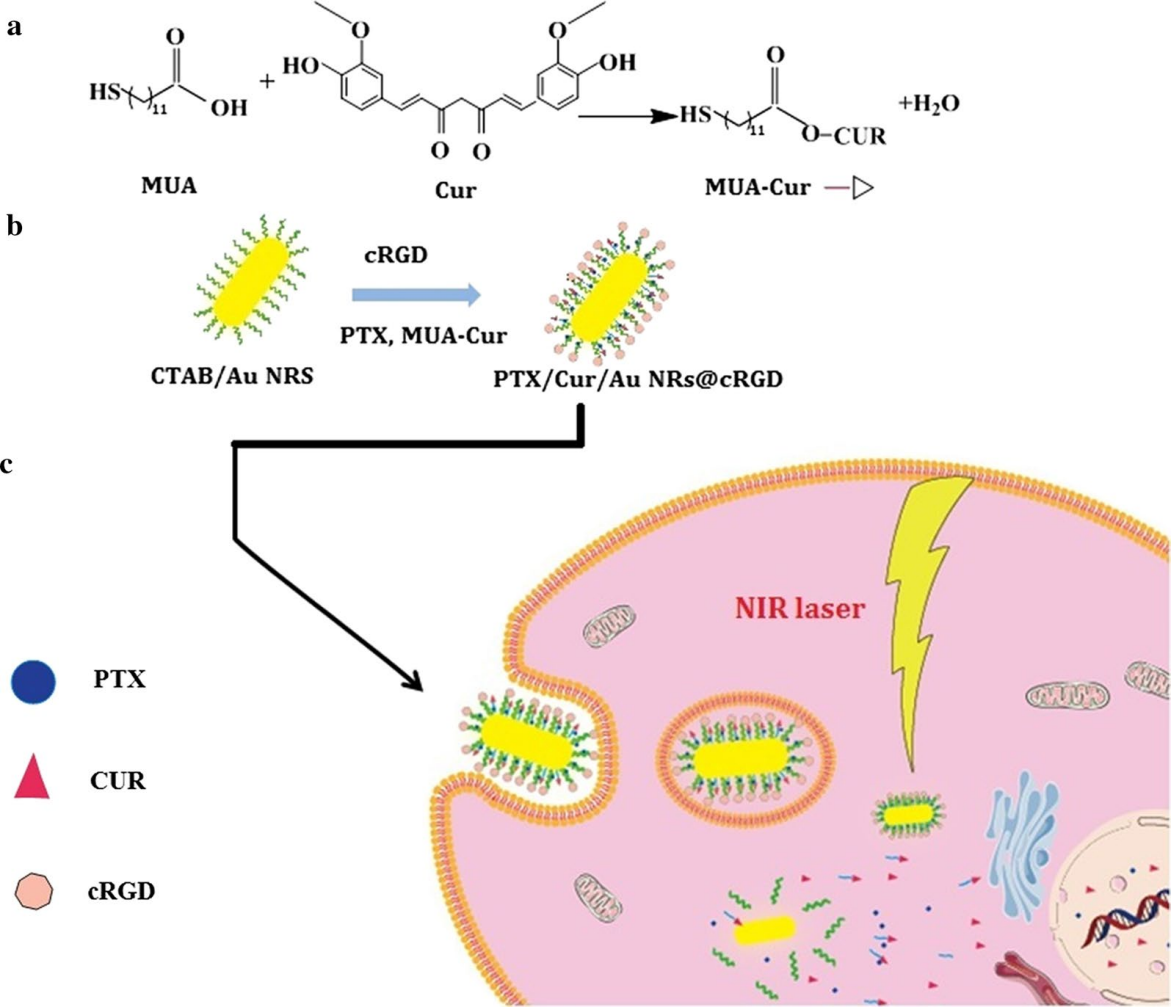
Schematic illustration of PTX/CUR/Au NRs@cRGD as novel various stimuli-responsive theranostic nanoplatforms for cancer treatment

To address these challenges, we designed a tumor-targeted and multi-stimuli-responsive Au NRs for the combination of PPTT and chemotherapy, which was modified by cRGD peptides and simultaneously delivered both the paclitaxel and curcumin with independent release manner (designated as PTX/CUR/Au NRs@cRGD) (Fig. 1). To prepare the nanocarrier, thiol-end-functionalized curcumin conjugates were synthesized by covalently linking 11-mercaptoundecanoic acid (MUA) to curcumin (MUA–Cur) via an ester bond. MUA–Cur and thiol end functionalized PEG was both modified on the Au NRs by replacing the cetyltrimethylammonium bromide (CTAB) on the surface of Au NRs. PTX was loaded in the hydrophobic alkanethiol-conjugated poly(ethylene glycol) (PEG) monolayer. The curcumin was supposed to be released as the ester bonds of MUA–Cur dissociated in the esterase-enriched tumor microenvironments. The PTX was cellular delivered through partitioning of the drug within the lipophilic plasma membrane. The cRGD peptides specifically bind to the *αvβ3* integrin receptors over-expressed on tumor cells, thus, to enhance the tumor cell uptake of the therapeutics. Moreover, the NIR laser irradiation is applied for photothermal therapy and further control the release of residual PTX and CUR on-demand. Based on these hypotheses, this unique PTX/CUR/Au NRs@cRGD was believed to exhibit a superior stability and improved on site drug delivery without a premature dose reduction under physiological conditions, and integrate the advantages of the active-targeting, photo-chemotherapy and controlled drug release. We systematically investigated the in vitro evaluation including presupposition, drug release, cell internalization, tumor cell cytotoxicity; and in vivo antitumor efficacy and safety of PTX/CUR/Au NRs@cRGD. The results exhibited a sufficient ablation of tumors via the synergistic effect and demonstrated that PTX/CUR/Au NRs@cRGD might be a promising candidate as a nanocarrier for chemophotothermal synergistic cancer therapy.

## Materials and methods

### Materials

The chemicals used to prepare the gold nanorods were all purchased from Sigma-Aldrich (St Louis, MO, USA). Dual functional poly(ethyleneglycol) (NH_2_–PEG–COOH, MW 5000 Da) and mPEG–NH_2_ (MW 5000 Da) were obtained from Laysan Bio (Arab, AL, USA). Paclitaxel was a product of Civi chemical technology Co., Ltd. (Shanghai, China). Curcumin was obtained from TCI-Chemical industry (Tokyo, Japan). c(RGDyK) was purchased from the ChinaPeptides (Shanghai, China). Calcein-AM and propidium iodide (PI) were obtained from Sigma-Aldrich (St Louis, MO, USA). All other solvents and reagents were of analytical grade and used directly.

### Preparation and characterization of gold nanorods

Au NRs were synthesized in accordance with the seed-mediated growth method used in previous reports [36]. The Au NRs were characterized using the UV–Vis–NIR absorption profile from a spectrometer (Agilent 8453, Santa Clara, CA). The shape of the Au NRs was determined using transmission electron microscopy (TEM) (Tecnai G2 Spirit, Hillsboro, OR). The size and zeta potential of the Au NRs were measured using dynamic light scattering (DLS) (Malvern zetasizer Nano S90, Malvern, UK).

### Synthesis and characterization of MUA–Cur

In brief, MUA (1.091 g, 5 mM), curcumin (3.684 g, 10 mM), and 4-(dimethylamino) pyridine (DMAP) (0.305 g, 2.5 mM) were dissolved in 120 mL of anhydrous dichloromethane (DCM) and 15 mL of dimethyl formamide (DMF), followed by the dropwise injection of DCC (1.288 g, 6.25 mM) in 20 mL of anhydrous DCM. The mixture was reacted in an ice-bath for 2 h and then stirred for 5–6 h at room temperature. Thin-layer chromatography (TLC) was used to monitor the progress of the reaction. To remove the excess DCM, the reaction mixture was dried using a rotary evaporator until reaction completion. The crude product was purified through silica gel column chromatography with a gradient elution. A bright yellow final product was obtained by solvent evaporation and re-crystallization. ^1^H NMR analysis of the MUA–Cur verified that the MUA was successfully conjugated with the curcumin.

### Synthesis of MUA–PEG

MUA–PEG–COOH was synthesized by covalent bonding the amino group of bifunctional PEG (H_2_N–PEG–COOH) with the carboxylic groups of MUA. Briefly, MUA (1 mM), H_2_N–PEG–COOH (10 mg), and DMAP (0.1 mg) were dissolved in anhydrous DCM in the presence of DCC (0.8 mg) as a catalyst. The reaction mixture was stirred for 12 h in darkness, and the solvent was evaporated. To separate the excess organic precipitant, the residue was dissolved in deionized water and filtered through a 0.2 μm membrane filter, followed by the addition of DTT (1 mM) to reduce the thiol. After a 30 min reduction in the dark, the product was collected by centrifugation (8500 rpm, 20 min) with a centrifugal filter (MWCO 3000 Da, Millipore) and then washed with deionized water three times. mPEG–MUA was prepared from the mPEG–NH_2_ and MUA using identical procedures, and the two lyophilized samples were characterized by ^1^H NMR.

### cRGD peptide targeting modification

To stabilize the cRGD peptide modification system, the Au NRs were successively conjugated with two types of MUA–PEG (mPEG–MUA and MUA–PEG–COOH). Firstly, 0.2 mL of 1 mM mPEG–MUA was added to 10 mL of 0.5 nM Au NRs. After 10 min of stirring, the mixture was centrifuged (14,000 rpm) for 10 min and reconstituted in 10 mL of deionized water. Subsequently, 0.2 mL of 1 mM MUA–PEG–COOH was introduced and stirred for 1 h, followed by centrifugation (14,000 rpm, 10 min) and re-dispersal in an identical volume of deionized water, which formed a mixed layer of mPEG–MUA and MUA–PEG–COOH on the surface of Au NRs. The pendant carboxylic groups of MUA–PEG–COOH allowed the modification of the amino groups of the cRGD peptide. The PEG-modified Au NRs were activated by EDC (1 mg/mL) and NHS (50 μL) for 5 min; the activated Au NRs were added to 100 μL of cRGDyK peptide solution (0.5 μM in deionized water). The mixture was gently stirred for 2 h and collected by centrifugation (14,000 rpm, 10 min).

### Paclitaxel and MUA–Cur co-loading

For the dual drug loading, 12 μL of 5 mM PTX in DMSO and 100 μL of 10 mM MUA–Cur in DMSO were simultaneously added to 2 mL of 0.5 nM cRGD-modified Au NRs; the solution was stirred for 1 h in darkness. The final product (PTX/CUR/Au NRs@cRGD) was collected by centrifugation (14,000 rpm, 10 min) and was washed once with deionized water. The UV–Vis-NIR absorption profile, size and zeta potential were obtained using an UV–Vis–NIR absorption spectrometer and a DLS Malvern Zetasizer. The amount of PTX or CUR loaded in the PTX/CUR/AuNRs@cRGD was evaluated by HPLC (Shimadzu, LC-20 AT Japan) through a reverse-phase C_18_ column (4.6 mm × 150 mm, pore size 5 μm, Kromasil). The mobile phase of PTX was a mixture of acetonitrile:water (50:50, v/v) delivered at a flow rate of 1.0 mL/min. PTX was detected at 227 nm with an ultraviolet–visible detector. A mixture of acetonitrile/water containing 4% v/v of acetic acid was used as the mobile phase of CUR. The 0.8 mL/min flow rate and a wavelength of 430 nm were set to test the CUR loading efficiency. To determine the surface density of cRGD peptides bound on the PTX/CUR/Au NR@cRGD, we measured the amounts of cRGD through BCA assay. Based on the manufacturer’s protocols, the reagent A was mixed with reagent B as BCA working solution, then, 1 nM PTX/CUR/Au NR@cRGD were added into 96-well plates, followed by cultured with 200 μL BCA working solution at 37 °C. The assay was performed at 570 nm using a microplate reader after 30 min co-incubated. DiR/PEG/Au NRs@cRGD or DiR/PEG/Au NRs were prepared through the identical procedures, DiR were used to replace PTX and without addition MUA–Cur. DiR is one of NIR responsive fluorescent probe to be used as marker for bio-distribution of DDSs.

### Drug release in vitro

The release of MUA–Cur from the PTX/CUR/Au NRs@cRGD was measured in the presence of different concentrations of esterase. PTX/CUR/Au NRs@cRGD in water was mixed with esterase in the tube, and the release mixture was kept in the dark at 37 °C. At every given time, the tubes were centrifuged (2500 rpm) for 5 min, and the supernatants were collected to analyze the amounts of CUR and PTX released using HPLC. PTX/CUR/Au NRs@cRGD was gently introduced into an identical volume of DCM. After the specified time intervals, the top DCM phase was collected, and the PTX in the organic solution was separated by centrifugation. To evaporate the solvent, the residue was re-dispersed in methanol and measured by HPLC to estimate the cumulative percentages of PTX and CUR that had been released.

### Cell culture

HepG2, KB and A549 cells were all purchased from American Type Culture Collection (ATCC) (Manassas, USA). The HepG2 and KB cells were maintained in DMEM medium, and the A549 cells were cultured in RPMI 1640 medium. The above medium was supplemented with 10% (V/V) FBS, 100 U/mL penicillin, and 100 U/mL streptomycin. The cells were incubated at 37 °C and a humidified atmosphere containing 5% CO_2_.

### Intracellular uptake

For the intracellular uptake studies, the A549 cells were incubated with PEG/Au NRs, PTX/CUR/Au NRs or PTX/CUR/Au NRs@cRGD for 3, 6, and 12 h. Then, the experimental medium was removed and the cells were washed three times with PBS. The treated cells were digested with trypsin including EDTA, centrifuged, and harvested as pellets. These cell pellets were fixed in 2.5% fixation glutaraldehyde, dehydrated, embedded and cut, followed by TEM observation with a transmission electron microscope (Hitachi H-7500, Japan) at 80 kV.

Confocal microscopic studies were carried out to detect the in vitro cellular uptake of PTX/CUR/Au NRs@cRGD and PTX/CUR/Au NRs in A549 cells. Briefly, A549 cells were cultured overnight on glass bottom dishes and treated with PTX/CUR/Au NRs@cRGD or PTX/CUR/Au NRs (CUR concentrations: 3.125 μM, 650 pM with respect to Au NRs concentration) for 6 h at 37 °C. Then, the cells were washed with PBS. The fluorescence was visualized using a confocal laser scanning microscopy (Olympus FV1000, Tokyo, Japan).

### Therapeutic efficacy in vitro

The A549, HepG2 and KB cells (8 × 10^3^ cells/well) were seeded in 96-well plates and allowed to adhere at 37 °C for 24 h. Then, the seeding medium was removed and added to the experimental medium containing various concentrations of Au NRs CUR/Au NRs, PTX/Au NRs, PTX/CUR/Au NRs and PTX/CUR/Au NRs@cRGD (25–400 pM, with respect to Au NRs concentration). After incubation, the cell viability was evaluated by cck-8 assay. To determine the effects of combining chemotherapy and PPTT, A549, HepG2 or KB cells were seeded in 96-well plates and cultured with 100 μL of an experimental medium that contained equivalent concentrations of PTX/CUR/Au NRs, PEG/Au NRs or PTX/CUR/Au NRs@cRGD. After 24 h of incubation, the PEG/Au NRs and PTX/CUR/Au NRs@cRGD groups were irradiated with a NIR laser (λ = 808 nm; beam size, 5 mm; power intensity, 0.55–0.7 W cm^−2^) (Diode laser, Changchun New Industries Optoelectronics Tech. Co. Ltd, China) for 10 min. The laser treatment cells were further incubated for 1 h with the drug-containing medium, after which every experimental group of cells was washed using PBS and incubated in fresh medium for 48 h. The cell viability was also determined through the cck-8 assay. An annexin V-FITC apoptosis detection kit was used to assess the apoptosis of three types of cancer cells after incubation with nanocarrier. The cell cycle arrest was performed using a cell cycle detection kit. According to the standard protocol, the A549, HepG2 and KB cells were incubated with the CUR/Au NRs, PTX/Au NRs, PTX/CUR/Au NRs and PTX/CUR/Au NRs@cRGD. Subsequently, PBS was used to wash the cells and cooled 70% ethanol was added to fix the cells for 4 h. To remove the ethanol through centrifugation, RNase and propiduium iodide were introduced into the fixed cells. The percentages of cells in the different phases were examined using a flow cytometer.

### In vivo NIRF imaging

To evaluate whether the nanocarrier targeted the tumor, in vivo NIRF imaging was performed. BALB/c nude mice bearing A549 tumors (female, 20–25 g) were injected with 200 µL of DiR/PEG/Au NRs@cRGD or DiR/PEG/Au NRs (2 nM) through the tail vein. The NIRF imaging of mice was monitored using a small-animal imaging system with an excitation wavelength of 780 nm at 2, 4, 6 and 8 h after injection. Then, the mice were sacrificed, and the bio-distributions of DiR/PEG/Au NRs@cRGD or DiR/PEG/Au NRs were investigated and imaged using the removed tumor tissues and major organs (heart, liver, spleen, lungs and kidneys).

### In vivo antitumor efficacy

All animal procedures were in compliance with the China Council on Animal Care and Use protocol. The experimental procedures were performed in accordance with the Guidelines for the Institutional Animal Care and Use Committee of Southern Medical University (Guangzhou, China) and approved by the Animal Ethics Committee of Southern Medical University. To examine the antitumor effect of PTX/CUR/Au NRs@cRGD in vivo, a tumor xenograft animal model was established. BALB/c nude mice bearing an A549 tumor were randomized and assigned into five groups, with five mice in every group. When the tumor volume increased to ~ 120 mm^3^, the five groups were injected with 200 µL of PBS, the 200 µL mixture of MUA–Cur and PTX (dissolved in DMSO and diluted with PBS, 0.91 mg kg^−1^ for MUA–Cur, 1.00 mg kg^−1^ for PTX), PEG/Au NRs@cRGD or PTX/CUR/Au NRs@cRGD (200 μL, the same dosage of Au NRs 2.5 mg kg^−1^). The photothermal therapy groups were irradiated for 5 min with a NIR laser (0.95 W cm^−2^) at 24 h after incubation with the nanocarrier. The tumor size was observed using a caliper and the body weights were measured every day for the following 15 days. The tumor volumes were calculated as tumor volume = (tumor length) × (tumor width)^2^/2. At the end of treatment, the mice were sacrificed, and the tumors and organs (heart, liver, spleen, lungs and kidneys) were removed. To evaluate the biocompatibility of the nanocarrier, the collected tissues, including the tumors, were washed using cold PBS, fixed with the formalin, and embedded in paraffin, H&E stain and observations using an optical microscope (Olympus IX71, Japan) were performed.

### Statistical analysis

All data are expressed as the mean ± standard deviation. A one-way ANOVA followed by Dunnett’s multiple comparison test and a paired t-test were used to analyze the statistical comparisons. A statistically significant difference was denoted when the *P* value was less than 0.05.

## Results and discussion

### Preparation and characterization of PTX/CUR/Au NRs@cRGD

Based on the seed-mediated growth method, Au NRs were obtained and characterized using the UV absorption spectrum (Additional file 1: Figures S1A, S1B). The prepared Au NRs possessed a localized surface plasmon resonance (SPR) peak around 780 nm and an aspect ratio of 3.7. The Au NRs had an average size of 55 nm × 15 nm and a positive charge of +38 mV because of the cationic surfactant coating (i.e., CTAB). The successful formation of the MUA–Cur and MUA–PEG–COOH was confirmed by ^1^H NMR analysis. The ^1^H spectra of the MUA–Cur showed the characteristic peak of curcumin at 9.83 ppm, doublet peaks at 3.82 ppm and the peak of MUA at 1.29 ~ 1.34 ppm (Additional file 1: Figure S1C). The typical ^1^H NMR peaks of MUA–PEG–COOH are ascribed to a combination of those of MUA and H_2_N–PEG–COOH, showing the successful conjugation of MUA and H_2_N–PEG–COOH (Additional file 1: Figure S1D).

**Fig. 2:**
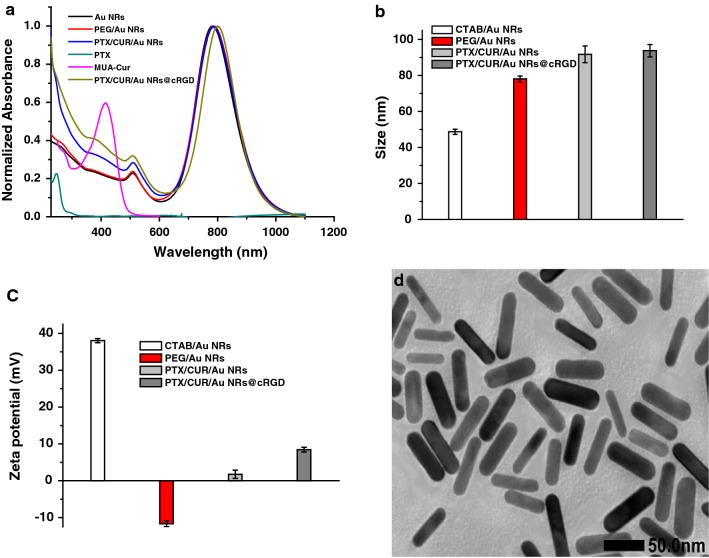
Characterization of Au NRs. **a** UV–Vis-NIR absorption profiles of Au NRs, PTX, MUA–Cur, PEG/Au NRs before and after cRGD coating. **b** Hydrodynamic diameters of CTAB/Au NRs, PEG/Au NRs, PTX/CUR/Au NRs and PTX/CUR/Au NRs@cRGD. **c** Zeta potential of CTAB/Au NRs, PEG/Au NRs, PTX/CUR/Au NRs and PTX/CUR/Au NRs@cRGD. **d** TEM analysis of PTX/CUR/Au NRs@cRGD

For biomedical applications, Au NRs were functionalized with two types of ligands to replace the CTAB, the MUA–Cur and MUA–PEG–COOH were successively anchored onto the Au NRs by thoilated linkers, due to the high affinity of gold for thiols. The MUA–Cur and MUA–PEG–COOH could form a hydrophobic region on the surfaces of Au NRs, which facilitated the PTX loading. Figure 2a displayed the absorption profiles of Au NRs, PTX, MUA–Cur, PEG/Au NRs, PTX/CUR/Au NRs and PTX/CUR/Au NRs@cRGD. Compared to PEG/Au NRs, the PTX/CUR/Au NRs had additional absorption peaks at 232 nm and 416 nm from the PTX and MUA–Cur, respectively, thus confirming the success of dual drug loading. The pendant carboxylic group of MUA–PEG–COOH allowed the covalent attachment of the amino groups of cRGD for surface modification, thereby improving the in vivo behavior of PTX/CUR/Au NRs@cRGD. Consequently, the absorption profile of PTX/CUR/Au NRs@cRGD indicated a 12 nm red shift (from 772 to 784 nm) of the original basis. The red shift of the SPR band of PTX/CUR/Au NRs@cRGD after being functioned with cRGD was attributed to the variety dielectric constant of the surroundings of the Au NRs. To verify successful surface modifications, the size and zeta potentials of the Au NRs at the different functional stages were also measured. The DLS histograms of PEG/Au NRs, PTX/CUR/Au NRs and PTX/CUR/Au NRs@cRGD had average particle size of 78.08 ± 1.64, 91.74 ± 4.66 and 93.76 ± 3.43 nm, respectively (Fig. 2b). The average size of the Au NRs (46.68 ± 0.42 nm) increased 30 nm when conjugated with the MUA–PEG–COOH, and was further augmented by 15 nm after cRGD coating. The hydrodynamic size distribution curve and PDI value of each nanoparticle (CTAB/Au NRs, PEG/Au NRs, PTX/CUR/Au NRs and PTX/CUR/Au NRs@cRGD) were shown in Additional file 1: Figure S2. When the PEG replaced CATB to stabilize the Au NRs, the particle dispersity decreased a little. After the PTX/CUR/Au NRs modification with cRGD, the PTX/CUR/Au NRs@cRGD was about 0.15 smaller than their PTX/CUR/Au NRs in the particle dispersity. These results indicate that the hydrophilic PEG exterior and cRGD modification make the system physiologically stable. The zeta potential of the PEG/Au NRs, PTX/CUR/Au NRs and PTX/CUR/Au NRs@cRGD were determined to be − 11.70, 1.75, and 8.42 mV, respectively (Fig. 2c). The system firstly underwent the reversion of the charge after the MUA–PEG–COOH modification, while the zeta potential returned to positive after conjugating with cRGD. Together, the absorption spectrum, DLS and zeta potential results were compelling proof of the successful preparation of the nanocarrier. Compare to Au NRs, TEM of PTX/CUR/Au NRs@cRGD presented no apparent changes after modification (Fig. 2d). Additionally, it has been reported that the thiol/disulfide redox couple can coexist on the gold nanoparticles surface and is favored by the disulfide formation for the high-density gold surface coating [37]. The loading efficiency of PTX and MUA–Cur were 7.6% (wt%) and 16.43% (wt%), respectively, examined by HPLC (Additional file 1: Figure S3). It was found that 4.56 × 10^3^ PTX and 9.85 × 10^4^ CUR was simultaneously loaded per Au NR in the system of PTX/CUR/Au NRs@cRGD (The detailed calculation of the number of drug per Au showed in Additional file 1). We measured the surface density of cRGD peptides and the results indicated that cRGD peptides efficiency was 37.3%.

### Controlled drug release and cellular uptake in vitro

**Fig. 3:**
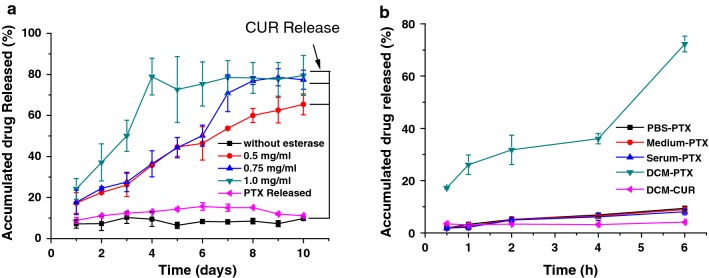
The cumulative drug release profiles under different release manner. **a** The cumulative release of curcumin and paclitaxel from PTX/CUR/Au NRs@cRGD in the presence of different concentrations of esterase. **b** The cumulative release profile of PTX and CUR from PTX/CUR/Au NRs@cRGD

Based on our design conception, the release behaviors of PTX and CUR from PTX/CUR/Au NRs@cRGD should be independent and have no influence on each other. To validate this hypothesis, we evaluated the PTX or CUR release under different conditions. The CUR release was examined in the presence of esterase due to the covalent bonding of MUA–Cur, and PTX was expected to exhibit no significant release in the same situation. For different concentrations of esterase, the release curves of CUR presented different release efficacies; a high concentration esterase in the tumor microenvironment resulted in an increased drug release [38, 39]. The CUR release achieved a plateau and kept a steady and slow release after the rapid release for the first 4 days (shown in Fig. 3a). In contrast, no obvious PTX was delivered under the CUR release condition. As a hydrophobic drug, the PTX in our system provides an opportunity for release into the cell via the detachment of the drug within the lipophilic plasma membrane [40]. To imitate the lipid membrane, the PTX release was estimated using a water/DCM two-phase system; the PTX was progressively released from the PTX/CUR/Au NRs@cRGD and assumed a clear bi-phase profile (Fig. 3b). After 6 h of exposure to DCM, more than 70% of the loaded PTX was released, and a trace amount of Au NRs appeared in the DCM, indicating an efficient release. We also observed that almost no CUR (< 5%) was released from the PTX/CUR/Au NRs@cRGD without the esterase over time, demonstrating that the loaded dual drugs followed different release patterns, with the CUR relying on enzyme hydrolysis and the PTX on hydrophobic interactions (Additional file 1: Figure S4). To access whether the temperature increase induced by light exposure on Au NRs accelerate the drug release, we additionally examined the drug release curve under light irradiation (Additional file 1: Figure S5). It was found that progressive release of CUR from the PTX/CUR/AuNRs@cRGD closed to 85% at the fifth days under laser exposure, the laser irradiation facilitated the PTX release, the accumulated drug release was 94% at 6 h. The results suggested the NIR further accelerated the drug release and enhanced chemotherapeutical efficiency.

**Fig. 4:**
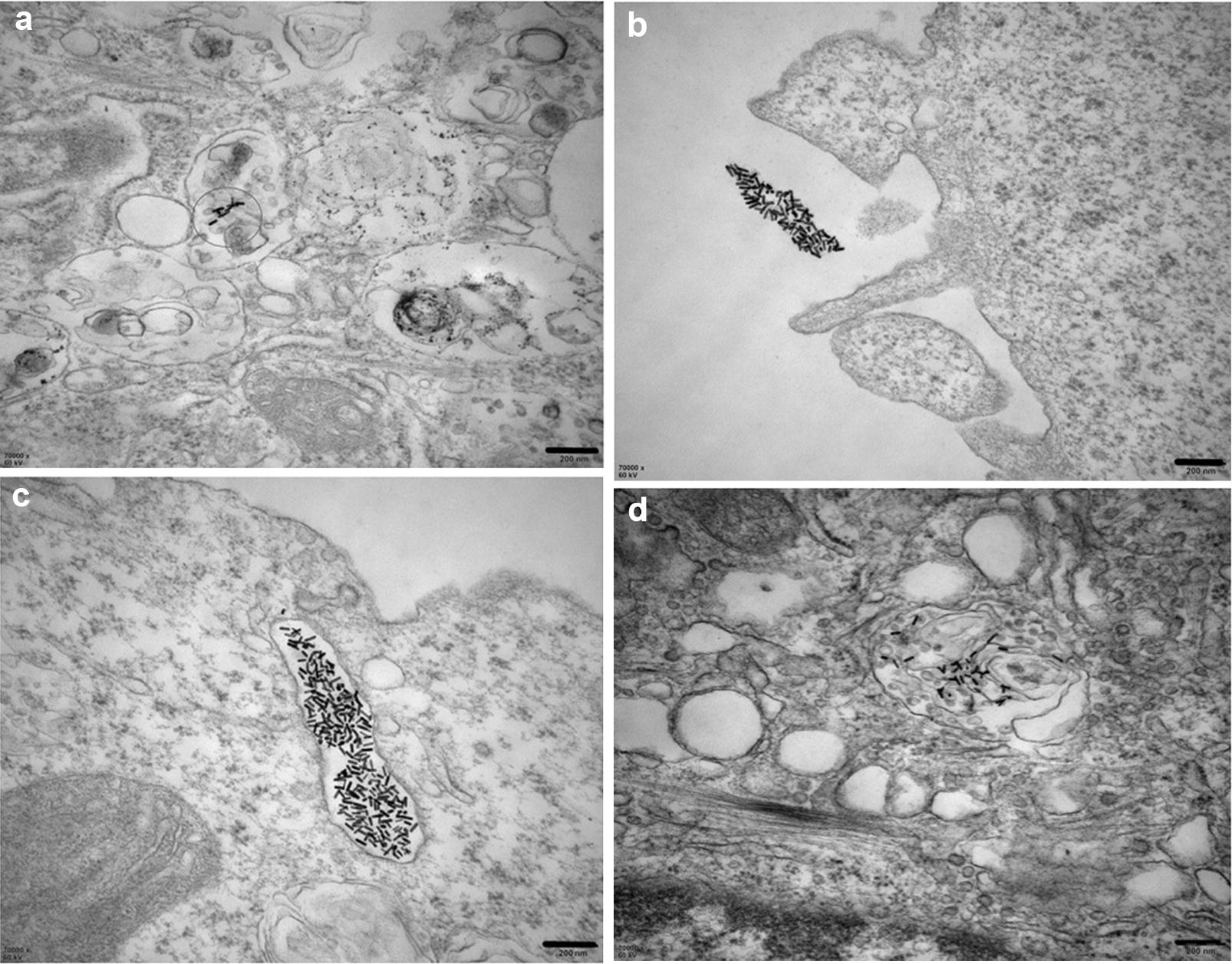
TEM images show the localization of PTX/CUR/Au NRs or PTX/CUR/Au NRs@cRGD inside an A549 cell. **a** Cellular internalization of PTX/CUR/Au NRs inside the A549 cytoplasm. **b** Image of the clusters of PTX/CUR/Au NRs@cRGD located on the cell membrane. **c** Image of the clusters of PTX/CUR/Au NRs@cRGD located at the endosomes. **d** Cellular internalization of PTX/CUR/Au NRs@cRGD inside the A549 cytoplasm. Scale bar: 200 nm

As we know, intracellular uptake is an essential procedure for the subsequent stimuli-responsive drug release. The location of PTX/CUR/Au NRs@cRGD within the cells was observed by TEM imaging, A549 cells were chosen due to their over-expressing of *αvβ3* integrin receptor. No PEG/Au NRs particles were identified within the obtained images (data not shown), which might due to their negative surface charge. A small amount of PTX/CUR/Au NRs were found in the cytoplasm after 6 h co-incubation. These observations agreed with the increased uptake of PTX/CUR/Au NRs by A549 lung epithelial cells upon intravascular injection (Fig. 4a) [41]. Interestingly, the clusters of PTX/CUR/Au NRs@cRGD both close to the cell membrane (Fig. 4b) and within the endosomal compartments (Fig. 4c) were observed after 6 h co-incubation, suggesting that the PTX/CUR/Au NRs@cRGD provided a relatively good colloidal stability, and subsequently enhanced the cellular uptake [42]. Moreover, intracellular PTX/CUR/Au NRs@cRGD could be easily found within the clear endosomal membrane, thus supporting endocytosis as a possible mechanism for the cellular uptake and in line with the literature data [43]. Additionally, a small fraction of PTX/CUR/Au NRs@cRGD appeared to be dispersed within the cytoplasm after 12 h co-incubation, which was an indicative of supplementary mechanisms of internalization, such as diffusion and endosomal release (Fig. 4d). When the A549 cells were pretreated with an excess of free cRGD peptides to retard *αvβ3* integrin, the uptake quantity of PTX/CUR/Au NRs@cRGD was significantly decreased, thus verifying that the intracellular uptake of PTX/CUR/Au NRs@cRGD was primarily reliant on the special target of the *αvβ3* integrin. The whole cell image was shown in Additional file 1: Figure S6, in which the cell membrane be apparently observed. To have a straightforward insight into the cellular uptake behavior of PTX/CUR/Au NRs@cRGD by multiple cells, we provide the confocal images in A549 cells. As shown in Additional file 1: Figure S7, stronger blue fluorescence signals from CUR was detected for PTX/CUR/Au NRs@cRGD in comparison with the PTX/CUR/Au NRs. It can be observed that PTX/CUR/Au NRs@cRGD or PTX/CUR/Au NRs distribute in the nucleoplasm. The modification with cRGD facilitate internalized by A549 cells which contain αvβ3 integrin receptor, while PTX/CUR/Au NRs present a certain amount of uptake. Recent reports showed that rod-shaped particles at the nanoscale have been exhibited to show higher cellular uptake and excellent ability of targeting in tumor xenografts [41, 44]. In the previous study, gold nanorods could lowered clearance of macrophage and further result in higher tumor accumulation [45, 46]. Therefore, our results coherently indicated that the rod-shaped particles (Au NRs) associated with greater internalization rates. The cellular uptake of PTX/CUR/Au NRs@cRGD was inhibited by cRGD pre-treated A549 cells.

### Chemotherapy and photothermal effects in vitro

**Fig. 5:**
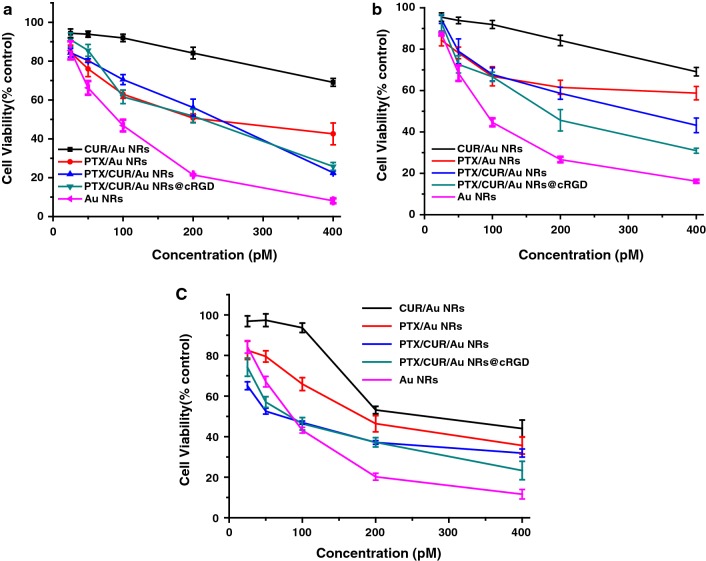
Chemotherapeutic effects of A549, HepG2 and KB tumor cells. Cell viability of **a** A549 cells, **b** HepG2 cells, **c** KB cells incubated with ACUR/Au NRs, PTX/Au NRs, PTX/CUR/Au NRs and PTX/CUR/Au NRs@cRGD for 48 h

The chemotherapy effect of Au NRs CUR/Au NRs, PTX/Au NRs, PTX/CUR/Au NRs and PTX/CUR/Au NRs@cRGD were examined on three cancer cell lines (A549, KB and HepG2) using a cck-8 assay. As shown in Fig. 5 and Additional file 1: Figure S8, cell viability exhibited a reduction in both time- and dose-dependent manner, and remarkable cytotoxicity was observed for incubation with PTX/CUR/Au NRs and PTX/CUR/Au NRs@cRGD. As expected,CUR/Au NRs showed mild cytotoxicity compare with the PTX/Au NRs, due to the former’s anticancer capacity and sustained release from the system. Notably, the dual drug-loaded system (PTX/CUR/Au NRs and PTX/CUR/Au NRs@cRGD) exhibited a higher cytotoxicity than the single drug-loaded system (CUR/Au NRs or PTX/Au NRs), despite the cell origins. To estimate whether the dual-drug-loaded system had the synergistic effects, we calculated the combination index (CI). The CI value was quantified using the following equation:

1 $${\text{CI}}\, = \,{{\text{a}} \mathord{\left/ {\vphantom {{\text{a}} {\text{A}}}} \right. \kern-0pt} {\text{A}}}\, + \,{{\text{b}} \mathord{\left/ {\vphantom {{\text{b}} {\text{B}}}} \right. \kern-0pt} {\text{B}}}$$

The equation where a and b are the IC_50_ of the PTX and CUR in the dual drug-loaded system and A and B are the IC_50_ of the PTX and CUR in the single drug-loaded system. CI values less than 1 meant synergism (Additional file 1: Table S1). The CI values for PTX/CUR/Au NRs@cRGD in the three cancer cell lines were all lower than 1, thus demonstrating that the enhanced cell mortality was ascribed to the synergistic effect of the CUR and PTX [31]. Therefore, PTX/CUR/Au NRs@cRGD was capable of achieving a high dual drug loading efficiency and synergistic effects of the combined drugs.

**Fig. 6:**
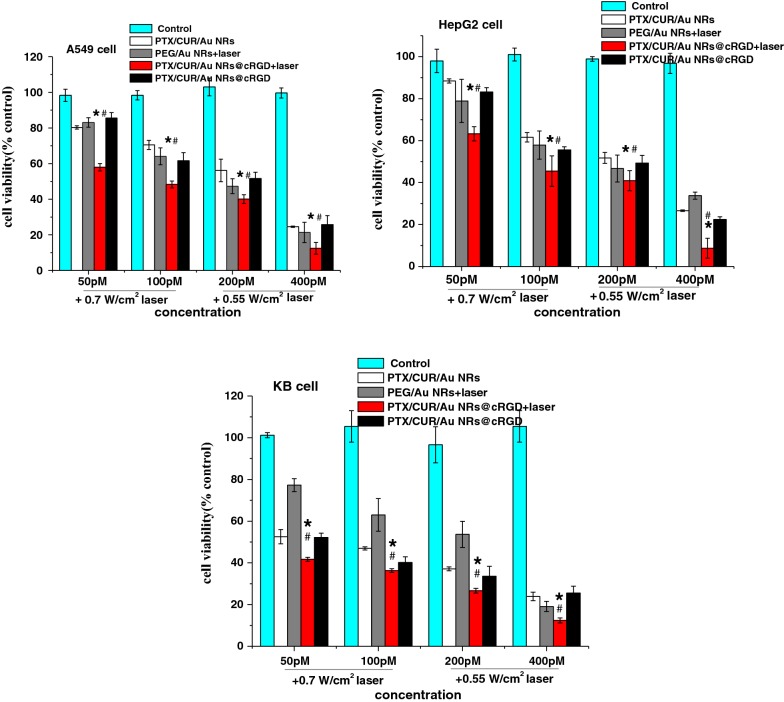
Combination therapy effects of the smart nanocarriers to the A549, HepG2 and KB tumor cell lines. PBS was used as a control. Data are presented as the mean ± SD (n = 5, *P < 0.05 vs PTX/CUR/Au NRs and ^#^*P *< 0.05 vs PEG/Au NRs + laser)

In light of the chemotherapy results, we investigated the effect of PTX/CUR/Au NRs@cRGD on the PPTT and combination therapy. As being known, the photothermal conversion efficiency of Au NRs (Additional file 1: Figure S9) should be assessed before PPTT therapy. The Au NRs, PBS and DMEM were irradiated with an 808 nm NIR laser at power densities of 0.5, 0.7 and 0.9 W cm^−2^. No obvious temperature elevation occurred for PBS and DMEM; whereas the temperatures of the Au NRs increased rapidly and evidently. After a 5 min exposure with laser irradiation at 0.7 W cm^−2^, the temperature of the Au NRs increased to 45 °C and reached a plateau, indicating that the as-prepared Au NRs presented an outstanding NIR light-responsive capacity, which encouraged us to perform the PPTT in vitro. For the PPTT therapy, three cancer cells were incubated with PEG/Au NRs or PTX/CUR/Au NRs@cRGD for 24 h, and exposed to the NIR laser for 5 min, and the cell viability was detected. In accordance with expectation, the high concentration of Au NRs and the increased illumination intensity exerted a severe cytotoxic effect. Attractively, the treatment with PTX/CUR/Au NRs@cRGD plus laser irradiation resulted in the strongest cytotoxicity among all the treatment groups (*P* < 0.05). The cell viability of PTX/CUR/Au NRs@cRGD was approximately 10%, implying excellent tumor ablation from the combined effect of the PPTT and chemotherapy (Fig. 6). We also evaluated the synergist effect index (Additional file 1: Table S2); and the results showed that a synergistic effect was found between the chemotherapy and PPTT regardless of the cell types or the concentration of Au NRs. In fact, in addition to the cell eradication due to the PPTT, an enhanced and controllable drug release as well as the chemo-sensitizer derived from hyperthermia also caused the cancer cell deaths [6].

To determine the possible mechanism of the combining therapy, we assessed the cell apoptosis and necrosis via flow cytometry analyses. Annexin V-FITC/PI double-stained cells were identified to and distinguish the apoptotic and necrotic cells. Compared with each single drug, chemotherapy with co-delivered drugs revealed enhanced cell apoptosis (Q2 + Q3) (A549: 71.95%; HepG2: 61.63%; KB: 64.34%), whereas the total apoptotic ratio of the combination treatment with PPTT and chemotherapy further increased to 82.88% (A549), 69.21% (HepG2), and 85.51% (KB) (Additional file 1: Figure S10). The above results clearly demonstrated that dual-therapy treatment could improve the therapeutic efficiency of PTX/CUR/Au NRs@cRGD by inducing more cell apoptosis. We also studied the underlying mechanism through the cell cycle distribution assay using flow cytometry. Compared to the single drug delivery system, the co-delivery system arrested more cells in the S phase. The number of S phase cells in the PPTT and chemotherapy treatment was calculated to be 56.02% (A549), 74.90% (HepG2) and 92.99% (KB), respectively, which were 31.23% (A549), 10.1% (HepG2) and 44.02% (KB) higher than those of the dual-drug chemotherapy treatments (Additional file 1: Figure S11). These results indicated that the PTX/CUR/Au NRs@cRGD together with the PPTT exhibited an extensive tumor cell-killing efficiency by S phase arrest in the three test cancer cell lines [47, 48].

### Tumor targeting and antitumor efficacy in vivo

**Fig. 7:**
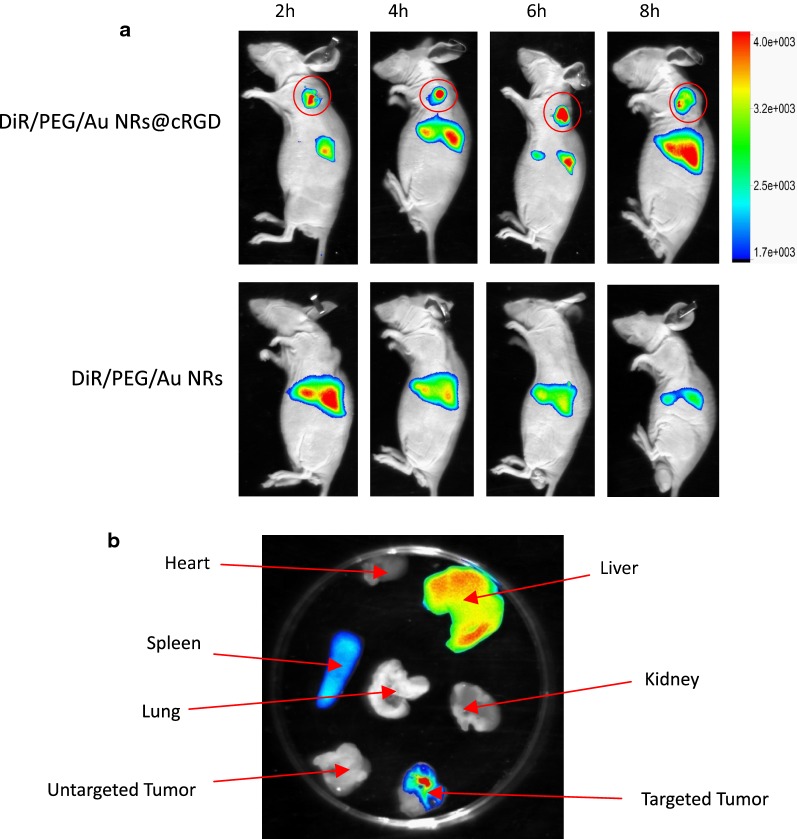
Representative NIRF images in vivo. **a** NIRF images of A549 tumor-bearing mice injected with DiR/PEG/Au NRs or DiR/PEG/Au NRs@cRGD at 2, 4, 6 and 8 h after injection (The semicircular region indicates tumor site). **b** The major organ distribution of DiR/PEG/Au NRs or DiR/PEG/Au NRs@cRGD in the subcutaneous A549 tumor model

To evaluate the in vivo behavior of PTX/CUR/Au NRs@cRGD, we established subcutaneous A549 tumor-bearing models. When the tumor volume reached ~ 120 mm^3^, 200 μL of DiR/PEG/Au NRs or DiR/PEG/Au NRs@cRGD in PBS was injected intravenously into the mice. We collected the time-dependent bio-distribution of the nanoparticles via a small animal in vivo imaging system. Figure 7a presents the images at different time points after the intravenous injection of the sample. Obviously, DiR/PEG/Au NRs@cRGD displayed a notable accumulation of near-infrared fluorescence (NIRF) signals at the tumors, and the signals generally weakened as time passed, whereas DiR/PEG/Au NRs exhibited no NIRF signals at the tumors. Interestingly, stronger fluorescence signals of DiR/PEG/Au NRs@cRGD were observed at tumor sites 2 h after injection compared with untargeted of DiR/PEG/Au NRs; and the fluorescence signals sequentially strengthened and achieved the highest level after 6 h injection. The increased tumor accumulation as well as retention favored the generation of elevated hyperthermia and chemotherapy efficacy. To estimate the relative tumor and organ distribution of DiR/PEG/Au NRs@cRGD, the major organs of the mice were harvested 2 days after injection, and the fluorescence signals of the organs were acquired (Fig. 7b). We found that the DiR/PEG/Au NRs@cRGD accumulated significantly in the tumor, liver and spleen, but did not distribute to the other organs. These results implied that DiR/PEG/Au NRs@cRGD could be stable in the bloodstream and rapidly target tumors without obvious damage to normal organs. Considering the possibility of the detachment of DiR from Au NRs, the quantitative bio-distribution of PTX/CUR/Au NRs@cRGD in vivo was estimated at 6 h post-injection through an ICP-MS analysis of gold content accumulated in the major tissues and tumor (Additional file 1: Figure S12). The results of PTX/CUR/Au NRs@cRGD exhibited that the accumulation of Au in the liver, spleen and tumors was much higher than those in the heart, lungs and kidneys, whereas Au levels of PTX/CUR/Au NRs were mostly present in the liver and spleen of mice. It was noteworthy that the uptake of PTX/CUR/Au NRs@cRGD in the tumor increased significantly compare with the PTX/CUR/Au NRs, likely due to the cRGD modification.

**Fig. 8:**
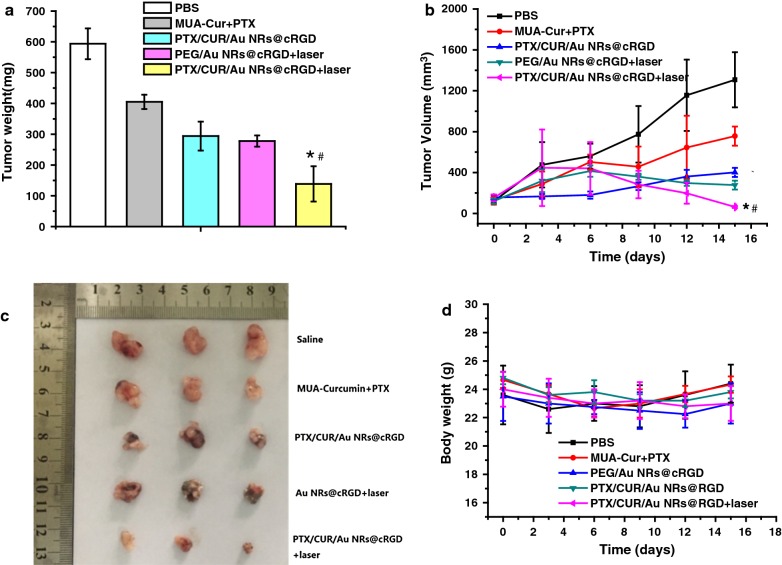
*In vivo* chemo-photothermal synergistic therapy in mice bearing a human lung cancer A549 cell xenograft. **a** Tumor weight changes of mice in the different groups. **b** Changes in tumor volume. Data are presented as the mean ± SD (n = 5, *P < 0.05 vs PTX/CUR/Au NRs@cRGD and ^#^*P* < 0.05 vs PEG/Au NRs@cRGD + laser). **c** Photograph of the excised tumors. **d** Body weight of A549-xenografted mice following a variety of treatments

We further examined the synergistic efficacy of PTX/CUR/Au NRs@cRGD in vivo. The mice bearing A549 tumors were injected with the mixture of MUA–Cur and PTX, PEG/Au NRs@cRGD or PTX/CUR/Au NRs@cRGD and saline as a control (Additional file 1: Figure S13). To combine the PPTT with the tumor chemotherapy, we irradiated the tumor using an 808 nm NIR laser for 5 min at power density of 0.95 W cm^−2^ after 24 h injection. The tumor volumes of every group were recorded during the subsequent 15 days to examine the antitumor effect. As seen in Fig. 8a, b, the PBS control group showed a 10-fold increase in tumor volume, indicating no suppression of tumor growth, and the simple mixture of MUA–Cur and PTX appeared poor anticancer potency. The inhibition of tumor growth in the photothermal treated mice (PEG/Au NRs@cRGD + laser, inhibition rate: 53.22%) was similar to the therapeutic effect of dual drug nano-chemotherapy (PTX/CUR/Au NRs@cRGD, inhibition rate: 50.50%), indicating that the single modal treatment had a comparable anticancer efficacy. It was noted that the PTX/CUR/Au NRs@cRGD group with NIR light irradiation significantly amplified the tumor ablation effects (inhibition rate: 76.67%) due to the combination of dual drug chemotherapy and photothermal therapy (Fig. 8c). Under mild laser exposure, PTX/CUR/Au NRs@cRGD possessed the highest anticancer efficacy among all therapy groups, indicating that the NIR and tumor microenvironment responsive of PTX/CUR/Au NRs@cRGD promoted the on-demand release and synergistic effects of PTX and CUR with NIR irradiation. Most importantly, PTX/CUR/Au NRs@cRGD presented an ideal tumor ablation efficacy, suggesting that NIR-light induced an enhancement of the dual drug delivery efficiency and resulted in a remarkable toxic effect toward cancer cells [49–53]. No obvious body weight loss was detected 15 days after the treatment, showing no significant organ damage or abnormalities in the mice treated by the PTX/CUR/Au NRs@cRGD (Fig. 8d). To evaluate the potential systemic toxicity of PTX/CUR/Au NRs@cRGD, we carried out histological examinations of the major organs via H&E staining (Additional file 1: Figure S14). The tumor tissue of the mice treated with combination therapy of PTX/CUR/Au NRs@cRGD and PPTT manifested a severe cancer apoptosis, cell shrinkage and nuclear damage, which led to the loose and fragile tumors. In contrast, in the mice treated with saline or the mixture of MUA–Cur and PTX, the tumor cells emerged with typical morphologies, such as large cell nucleus, which indicated the continuous proliferation of the tumors. No noticeable inflammatory lesions or organ damage were observed in the collected major organs, demonstrating the excellent biocompatibility and negligible side effects of the PTX/CUR/Au NRs@cRGD.

## Conclusions

In conclusion, for combination of chemotherapy and PPTT, we fabricated a nanocarrier based on gold nanorods, which was multiple stimuli-responsive for sequential dual drug release and specifically tumor targeted by cRGD peptide coating. Profiting from the tumor microenvironment-responsive character of the prepared nanocarrier and the intrinsic photothermal effects of Au NRs, the system possesses a dual-mode and independent drug release manner: CUR depends on enzyme hydrolysis, and PTX relies on hydrophobic interactions. A NIR light laser well controlled the release of the remainder of the drug. The coating of cRGD endowed the Au NRs with both enhanced tumor cellular internalization and negligible side effects to other normal organs. Along with the PPTT, synergetic anticancer effect in vitro and tumor ablation were observed in mouse models. This strategy integrates the chemo-photothermal effects, active tumor targeting, and multiple stimuli-responsive dual drug release to provide a versatile approach toward precise cancer nanotherapy.

## Additional file

**Additional file 1.** Additional figures and tables.

## References

[1] Nam J, La WG, Hwang S, Ha YS, Park N, Won N, Jung S, Bhang SH, Ma YJ, Cho YM, Jin M, Han J, Shin JY, Wang EK, Kim SG, Cho SH, Yoo J, Kim BS, Kim S (2013). pH-responsive assembly of gold nanoparticles and “spatiotemporally concerted” drug release for synergistic cancer therapy. ACS Nano.

[2] Yu M, Guo F, Wang J, Tan F, Li N (2016). A pH-driven and photoresponsive nanocarrier: remotely-controlled by near-infrared light for stepwise antitumor treatment. Biomaterials.

[3] Kim H, Kim Y, Kim IH, Kim K, Choi Y (2014). ROS-responsive activatable photosensitizing agent for imaging and photodynamic therapy of activated macrophages. Theranostics..

[4] Ling D, Park W, Park SJ, Lu Y, Kim KS, Hackett MJ, Kim BH, Yim H, Jeon YS, Na K, Hyeon T (2014). Multifunctional tumor pH-sensitive self-assembled nanoparticles for bimodal imaging and treatment of resistant heterogeneous tumors. J Am Chem Soc.

[5] Zhang J, Yuan ZF, Wang Y, Chen WH, Luo GF, Cheng SX, Zhuo RX, Zhang XZ (2013). Multifunctional envelope type mesoporous silica nanoparticles for tumor-triggered targeting drug delivery. J Am Chem Soc.

[6] Zhang L, Su H, Cai J, Cheng D, Ma Y, Zhang J, Zhou C, Liu S, Shi H, Zhang Y, Zhang C (2016). A multifunctional platform for tumor angiogenesis-targeted chemo-thermal therapy using polydopamine-coated gold nanorods. ACS Nano.

[7] Han HS, Choi KY, Lee H, Lee M, An JY, Shin S, Kwon S, Lee DS, Park JH (2016). Gold-nanoclustered hyaluronan nano-assemblies for photothermally maneuvered photodynamic tumor ablation. ACS Nano.

[8] Borri C, Centi S, Ratto F, Pini R (2018). Polylysine as a functional biopolymer to couple gold nanorods to tumor-tropic cells. J Nanobiotechnol.

[9] Zhang P, Wang C, Zhao J, Xiao A, Shen Q, Li L, Zhang J, Min Q, Chen J, Chen HY, Zhu JJ (2016). Near infrared-guided smart nanocarriers for microRNA-controlled release of doxorubicin/siRNA with intracellular ATP as fuel. ACS Nano.

[10] Patino T, Mahajan U, Palankar R, Medvedev N, Walowski J, Münzenberg M, Mayerle J, Delcea M (2015). Multifunctional gold nanorods for selective plasmonic photothermal therapy in pancreatic cancer cells using ultra-short pulse near-infrared laser irradiation. Nanoscale..

[11] Sperling RA, Rivera GP, Zhang F, Zanella M, Parak WJ (2008). Biological applications of gold nanoparticles. Chem Soc Rev.

[12] Lal S, Clare SE, Halas NJ (2008). Nanoshell-enabled photothermal cancer therapy: impending clinical impact. Acc Chem Res.

[13] Bagley AF, Hill S, Rogers GS, Bhatia SN (2013). Plasmonic photothermal heating of intraperitoneal tumors through the use of an implanted near-infrared source. ACS Nano.

[14] Huschka R, Barhoumi A, Liu Q, Roth JA, Ji L, Halas NJ (2012). Gene silencing by gold nanoshell-mediated delivery and laser-triggered release of antisense oligonucleotide and siRNA. ACS Nano.

[15] Yin J, Chen D, Wu S, Li C, Liu L, Shao Y (2017). Tumor-targeted nanoprobes for enhanced multimodal imaging and synergistic photothermal therapy: core-shell and dumbbell Gd-tailored gold nanorods. Nanoscale..

[16] Sun B, Wu J, Cui S, Zhu H, An W, Fu Q, Shao C, Yao A, Chen B, Shi D (2017). In situ synthesis of graphene oxide/gold nanorods theranostic hybrids for efficient tumor computed tomography imaging and photothermal therapy. Nano Res..

[17] Yang M, Liu Y, Hou W, Zhi X, Zhang C, Jiang X, Pan F, Yang Y, Ni J, Cui D (2017). Mitomycin C-treated human-induced luripotent stem cells as a safe delivery system of gold nanorods or targeted photothermal therapy of gastric cancer. Nanoscale..

[18] Li L, Chen C, Liu H, Fu C, Tan L, Wang S, Fu S, Liu X, Meng X, Liu H (2016). Multifunctional carbon-silica nanocapsules with gold core for synergistic photothermal and chemo-cancer therapy under the guidance of bimodal imaging. Adv Funct Mater.

[19] Conde J, Oliva N, Zhang Y, Artzi N (2016). Local triple-combination therapy results in tumour regression and prevents recurrence in a colon cancer model. Nat Mater.

[20] Tang XC, Tan LW, Shi K, Peng JR, Xiao Y, Li WT, Chen L, Yang Q, Qian Z (2018). Gold nanorods together with HSP inhibitor-ver-155008 micelles for colon cancer mild-temperature photothermal therapy. Acta Pharm Sin B..

[21] Xu X, Huang Z, Huang Z, Zhang X, He S, Sun X, Shen Y, Yan M, Zhao C (2017). Injectable, NIR/pH-responsive nanocomposite hydrogel as long-acting implant for chemo-photothermal synergistic cancer therapy. ACS Appl Mater Interfaces.

[22] Zhang Z, Wang L, Wang J, Jiang X, Li X, Hu Z, Ji Y, Wu X, Chen C (2012). Mesoporous silica-coated gold nanorods as a light mediated multifunctional theranostic platform for cancer treatment. Adv Mater.

[23] Zhang Z, Wang J, Nie X, Wen T, Ji Y, Wu X, Zhao Y, Chen C (2014). Near infrared laser-induced targeted cancer therapy using thermoresponsive polymer encapsulated gold nanorods. J Am Chem Soc.

[24] Li P, Shi YW, Li BX, Xu WC, Shi ZL, Zhou C, Fu S (2015). Photo-thermal effect enhances the efficiency of radiotherapy using Arg-Gly-Asp peptides-conjugated gold nanorods that target αvβ3 in melanoma cancer cells. J Nanobiotechnol.

[25] Duan X, Xiao J, Yin Q, Zhang Z, Yu H, Mao S, Li Y (2013). Smart pH-sensitive and temporal-controlled polymeric micelles for effective combination therapy of doxorubicin and disulfiram. ACS Nano.

[26] Cong Y, Xiao H, Xiong H, Wang Z, Ding J, Li C, Chen X, Liang X, Zhou D, Huang Y (2018). Dual drug backboned shattering polymeric theranostic nanomedicine for synergistic eradication of patient derived lung cancer. Adv Mater.

[27] Singla AK, Garg A, Aggarwal D (2002). Paclitaxel and its formulations. Int J Pharm.

[28] Gelderblom H, Verweij J, Nooter K, Sparreboom A, Cremophor EL (2001). The drawbacks and advantages of vehicle selection for drug formulation. Eur J Cancer.

[29] Heger M (2017). Drug screening: don’t discount all curcumin trial data. Nature.

[30] Toole MG, Soucy PA, Chauhan R, Raju MV, Patel DN, Nunn BM, Keynton MA, Ehringer WD, Nantz MH, Keynton RS, Gobin AS (2016). Release-modulated antioxidant activity of a composite curcumin-chitosan polymer. Biomacromolecules..

[31] Yang Z, Sun N, Cheng R, Zhao C, Liu Z, Li X, Liu J, Tian Z (2017). pH multistage responsive micellar system with charge-switch and PEG layer detachment for co-delivery of paclitaxel and curcumin to synergistically eliminate breast cancer stem cells. Biomaterials.

[32] Zhan Y, Chen Y, Liu R, Zhang H, Zhang Y (2014). Potentiation of paclitaxel activity by curcumin in human breast cancer cell by modulating apoptosis and inhibiting EGFR signaling. Arch Pharm Res..

[33] Yao Q, Gutierrez DC, Hoang NH, Kim D, Wang R, Hobbs C, Zhu L (2017). Efficient codelivery of paclitaxel and curcumin by novel bottlebrush copolymer-based micelles. Mol Pharm.

[34] Boztas AO, Karakuzu O, Galante G, Ugur Z, Kocabas F, Altuntas CZ, Yazaydin AO (2013). Synergistic interaction of paclitaxel and curcumin with cyclodextrin polymer complexation in human cancer cells. Mol Pharm.

[35] Ruttala HB, Ko YT (2015). Liposomal co-delivery of curcumin and albumin/paclitaxel nanoparticle for enhanced synergistic antitumor efficac. Colloids Surf B Biointerfaces..

[36] Nikoobakht B, El-Sayed MA (2003). Preparation and growth mechanism of gold nanorods (NRs) using seed-mediated growth method. Chem Mater.

[37] Li VA, Jimenez AD, Henriksen-Lacey M, Giammona G, Licciardi M, Liz-Marzán LM (2016). Inulin coated plasmonic gold nanoparticles as a tumor-selective tool for cancer therapy. J Mater Chem B..

[38] Wichitnithad W, Nimmannit U, Callery PS, Rojsitthisak P (2011). Effect of different carboxylic ester spacers on chemical stability, release characteristics, and anticancer activity of mono-PEGylated curcumin conjugates. J Pharm Sci.

[39] Zhu F, Tan G, Jiang Y, Yu Z, Ren F (2018). Rational design of multi-stimuli-responsive gold nanorod-curcumin conjugates for chemo-photothermal synergistic cancer therapy. Biomater Sci..

[40] Ren F, Bhana S, Norman DD, Johnson J, Xu L, Baker DL, Parrill AL, Huang X (2013). Gold nanorods carrying paclitaxel for photothermal-chemotherapy of cancer. Bioconjug Chem.

[41] Kolhar P, Anselmo AC, Gupta V, Pant K, Prabhakarpandian B, Ruoslahti E, Mitragotri S (2013). Using shape effects to target antibody-coated nanoparticles to lung and brain endothelium. Proc Natl Acad Sci USA.

[42] Brandenberger C, Mühlfeld C, Ali Z, Lenz AG, Schmid O, Parak WJ, Gehr P, Rothen-Rutishauser B (2010). Quantitative evaluation of cellular uptake and trafficking of plain and polyethylene glycol-coated gold nanoparticles. Small.

[43] Debrosse MC, Comfort KK, Untener EA, Comfort DA, Hussain SM (2013). High aspect ratio gold nanorods displayed augmented cellular internalization and surfacechemistry mediated cytotoxicity. Mater Sci Eng C Mater Biol Appl..

[44] Gratton SE, Ropp PA, Pohlhaus PD, Luft JC, Madden VJ, Napier ME, DeSimone JM (2008). The effect of particle design on cellular internalization pathways. Proc Natl Acad Sci USA.

[45] Park JH, von Maltzahn G, Zhang L, Schwartz MP, Ruoslahti E, Bhatia SN, Sailor MJ (2008). Magnetic iron oxide nanoworms for tumor targeting and imaging. Adv Mater.

[46] Barua S, Yoo JW, Kolhar P, Wakankar A, Gokarn YR, Mitragotri S (2013). Particle shape enhances specificity of antibody-displaying nanoparticles. Proc Natl Acad Sci USA.

[47] Ma N, Wu FG, Zhang X, Jiang YW, Jia HR, Wang HY, Li YH, Liu P, Gu N, Chen Z (2017). Shape-dependent radiosensitization effect of gold nanostructures in cancer radiotherapy: comparison of gold nanoparticles, nanospikes, and nanorods. ACS Appl Mater Interfaces.

[48] Yin F, Yang C, Wang Q, Zeng S, Hu R, Lin G, Tian J, Hu S, Lan RF, Yoon HS, Lu F, Wang K, Yong KT (2015). A light-driven therapy of pancreatic adenocarcinoma using gold nanorods-based nanocarriers for co-delivery of doxorubicin and siRNA. Theranostics..

[49] Ali MR, Rahman MA, Wu Y, Han T, Peng X, Mackey MA, Wang D, Shin HJ, Chen ZG, Xiao H, Wu R, Tang Y, Shin DM, El-Sayed MA (2017). Efficacy, long-term toxicity, and mechanistic studies of gold nanorods photothermal therapy of cancer in xenograft mice. Proc Natl Acad Sci USA.

[50] Xia Y, Wu X, Zhao J, Zhao J, Li Z, Ren W, Tian Y, Li A, Shen Z, Wu A (2016). Three dimensional plasmonic assemblies of Au NPs with an overall size of sub-200 nm for chemo-photothermal synergistic therapy of breast cancer. Nanoscale..

[51] Pérez-Hernández M, Del PP, Mitchell SG, Moros M, Stepien G, Pelaz B, Parak WJ, Gálvez EM, Pardo J, de la Fuente JM (2015). Dissecting the molecular mechanism of apoptosis during photothermal therapy using gold nanoprisms. ACS Nano.

[52] Ma M, Chen H, Chen Y, Wang X, Chen F, Cui X, Shi J (2012). Au capped magnetic core/mesoporous silica shell nanoparticles for combined photothermo-/chemo-therapy and multimodal imaging. Biomaterials.

[53] Wang X, Zhang J, Wang Y, Wang C, Xiao J, Zhang Q, Cheng Y (2016). Multi-responsive photothermal-chemotherapy with drug-loaded melanin-like nanoparticles for synergetic tumor ablation. Biomaterials.

