# Supplementary material for: Smart nanoplatform for sequential drug release and enhanced chemo-thermal effect of dual drug loaded gold nanorod vesicles for cancer therapy
Source: J Nanobiotechnology. 2019 Mar 27;17:44. doi: 10.1186/s12951-019-0473-3 (PMC6437988; doi:10.1186/s12951-019-0473-3)
Supplement: Supplementary file 1 — Additional file 1. Additional figures and tables. [file 12951_2019_473_MOESM1_ESM.docx]

**Additional file 1**

**Smart Nanoplatform for Sequential Drug Release and Enhanced Chemo-Thermal Effect of Dual Drug Loaded Gold Nanorod Vesicles for Cancer Therapy**

**Falian Zhu^1^, Guozhu Tan^1^, Yingtao Zhong^1^, Yaodong Jiang^2^, Lulu Cai^3^, Zhiqiang Yu^4^, Shuwen Liu^4,^*, and Fei Ren^1,^***

^1^ Department of Pharmacy, Nanfang Hospital, School of Pharmaceutical Sciences Guangdong Provincial Key Laboratory of New Drug Screening, Southern Medical University, Guangzhou 510515, China

^2^ Department of Urology, Nanfang Hospital, Southern Medical University,Guangzhou 510515, China

^3^ Personalized Drug Therapy Key Laboratory of Sichuan Province, Sichuan Academy of Medical Sciences and Sichuan Provincial People’s Hospital, School of Medicine, University of Electronic Science and Technology of China, Chengdu, Sichuan 610072, China

^4^ School of Pharmaceutical Sciences, Guangdong Provincial Key Laboratory of New Drug Screening, Southern Medical University, Guangzhou 510515, China

*Correspondence: [paper_mail@126.com](mailto:paper_mail@126.com%20) ; [liusw@smu.edu.cn](mailto:liusw@smu.edu.cn)


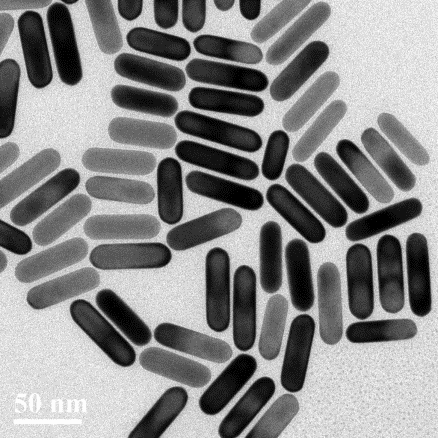


**b**


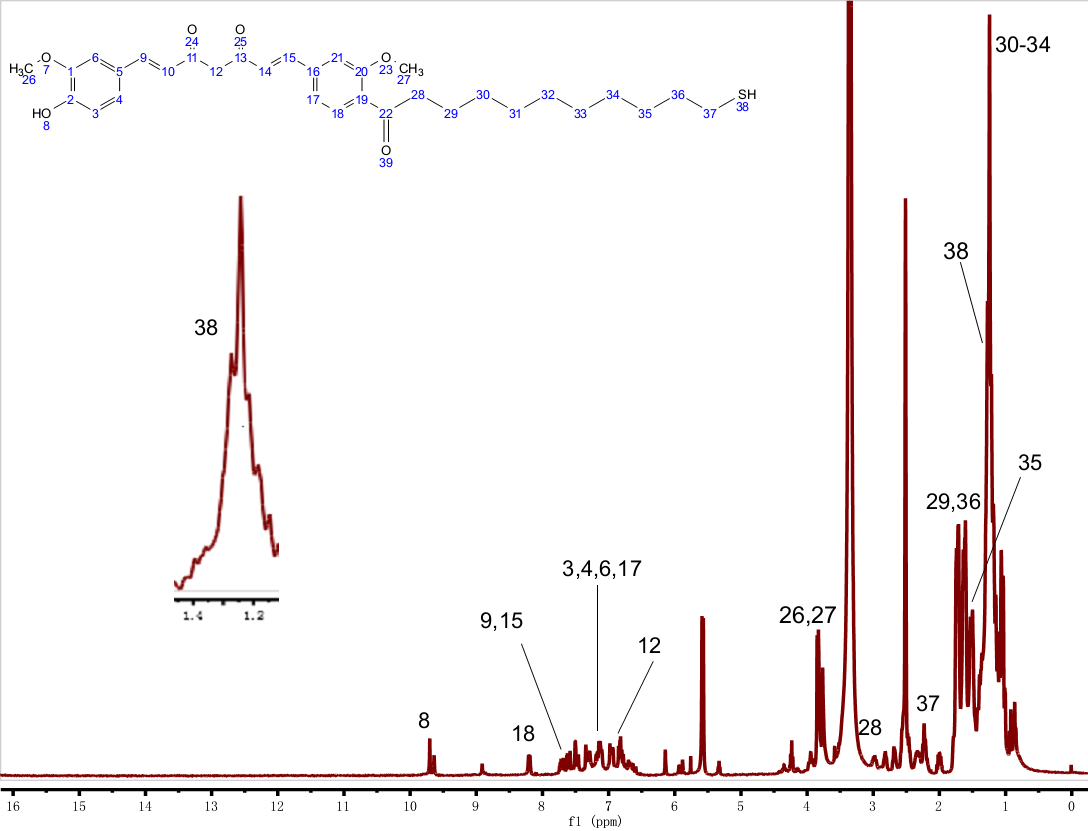


**C**


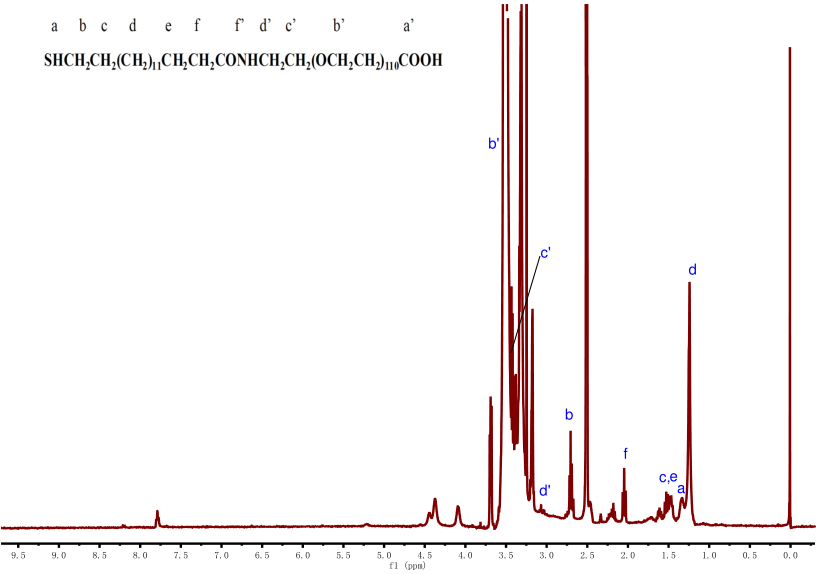


**d**

Additional file 1: Figure S1: **a** UV-Vis spectra of Au NRs coated with CTAB. **b** TEM micrograph of Au NRs. **c** ^1^H-NMR spectra of MUA-curcumin conjugates. **d** ^1^H-NMR spectra of MUA-PEG-COOH.

Additional file 1: Figure S2: Particle size distribution of CTAB/Au NRs, PEG/Au NRs, PTX/CUR/Au NRs and PTX/CUR/Au NRs@cRGD.

Additional file 1: Figure S3: HPLC chromatograms obtained from Au NRs, PTX/ Au NRs@cRGD, PTX and Au NRs, CUR/Au NRs@cRGD, CUR.

The number of drug per Au was calculated as drug loading capacity (%) × initial amount of drug/ amount of Au nanoparticles.

For CUR: 16.43 % × 0.3 mmol/ 0.5×10^-6^ mmol = 9.85 × 10^4^

Additional file 1: Figure S4: The curcumin release from PTX/CUR/Au NRs@cRGD in water/DCM solution and the stability of PTX/CUR/Au NRs@cRGD in PBS, cell culture medium, and serum.

Additional file 1: Figure S5: (a) Release profile of CUR from PTX/CUR/Au NRs@cRGD with 0.1 mg/mL esterase and in response to NIR laser irradiation (0.75 W/cm^-2^). (b) The cumulative release profile of PTX from PTX/CUR/Au NRs@cRGD in response to NIR laser irradiation (0.75 W/cm^-2^).


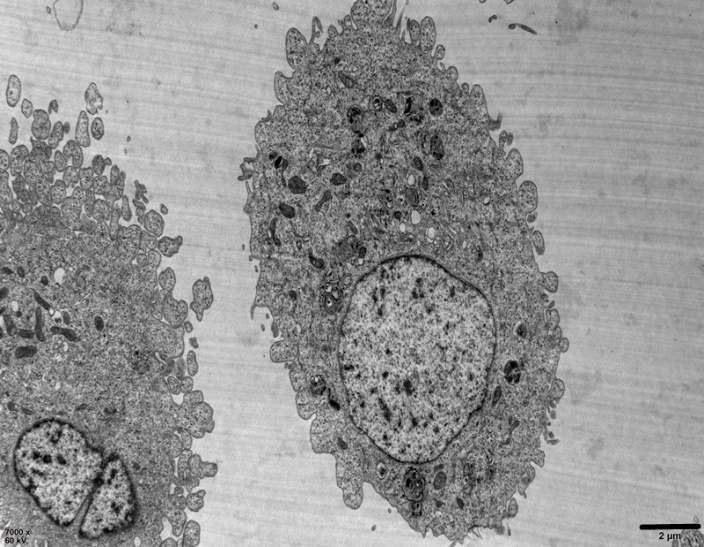


Additional file 1: Figure S6: The whole A549 cell image by TEM. Scale bar: 2 µm.

Bright field Curcumin Merge


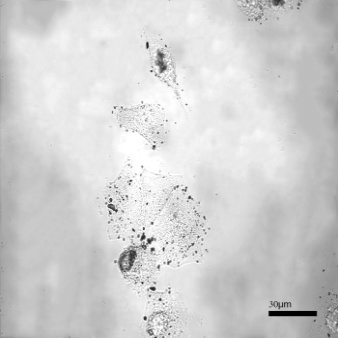

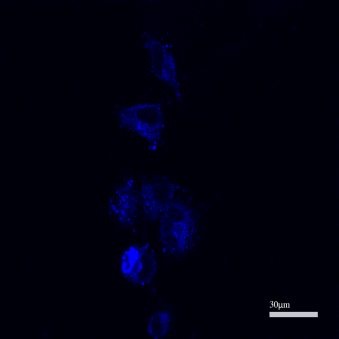

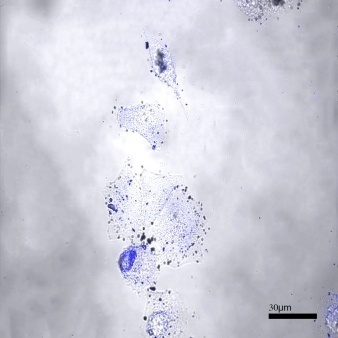


PTX/CUR/Au NRs@cRGD


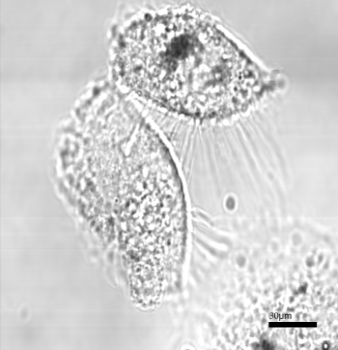

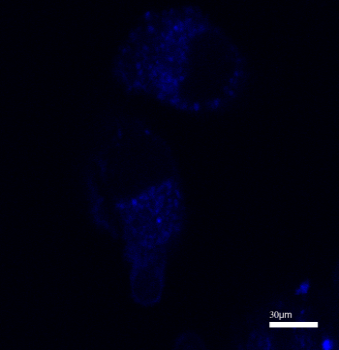

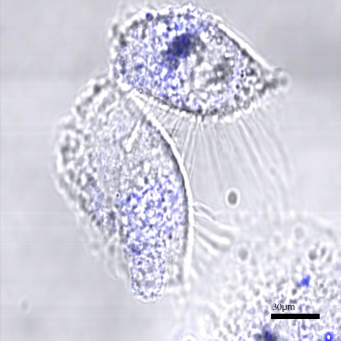


PTX/CUR/Au NRs


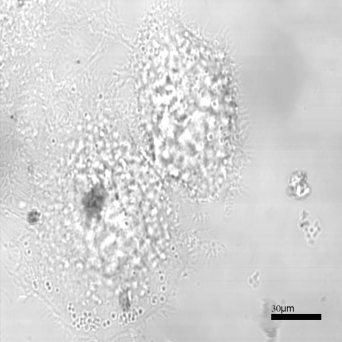

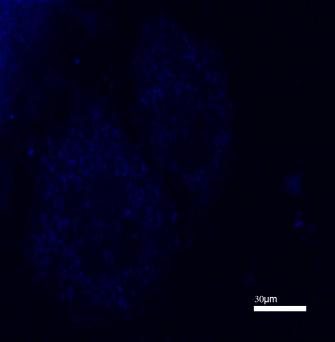

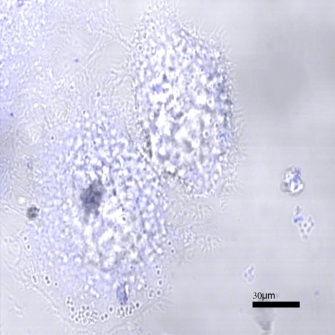


Blocking cRGD

Additional file 1: Figure S7: Confocal laser scanning microscopic (CLSM) images of A549 cells treated with PTX/CUR/Au NRs@cRGD, PTX/CUR/Au NRs and pre-treated with free cRGD before co-incubation of PTX/CUR/Au NRs@cRGD for 6 h. From left to right, images present bright field, the blue fluorescence of curcumin was analyzed by a confocal microscope. Bars represent 30 μm.

Additional file 1: Figure S8: Cell viability of A549 cells incubated with Au NRs, CUR/Au NRs, PTX/Au NRs, PTX/CUR/Au NRs and PTX/CUR/Au NRs@cRGD for **a** 24 h and **b** 72 h, HepG2 cells incubated with Au NRs, CUR/Au NRs, PTX/Au NRs, PTX/CUR/Au NRs and PTX/CUR/Au NRs@cRGD for **c** 24 h and **d** 72 h, KB cells incubated with Au NRs, CUR/Au NRs, PTX/Au NRs, PTX/CUR/Au NRs and PTX/CUR/Au NRs@cRGD for **e** 24 h and **f** 72 h.

Additional file 1: Figure S9: Temperature changes of Au NRs after NIR laser (808 nm) irradiation at 0.5, 0.7 or 0.9 W cm^-2^ for 5 min.

PTX/CUR/

AuNRs@cRGD

PTX/CUR/AuNRs@cRGD+ laser

Cur/Au NRs

PTX/Au NRs

Control

**
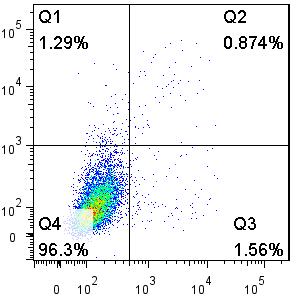
** **
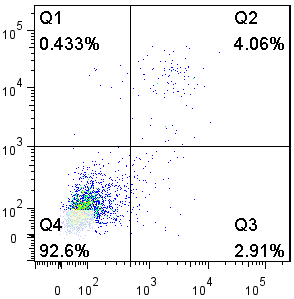
** **
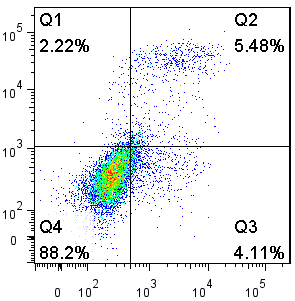
** **
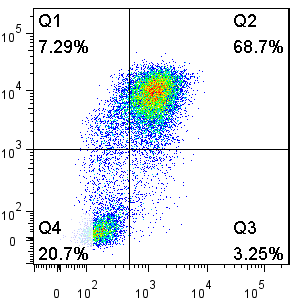
** **
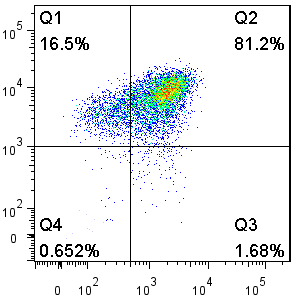
**

A549

**
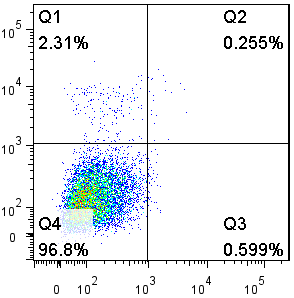
** **
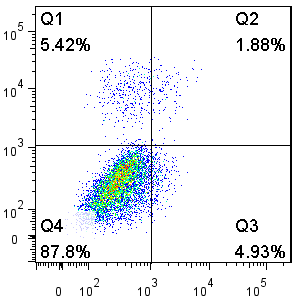
** **
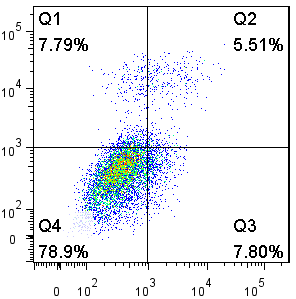
** **
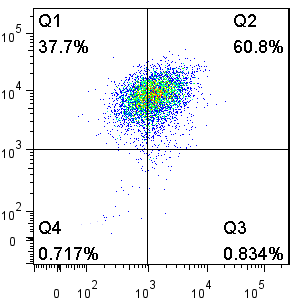
** **
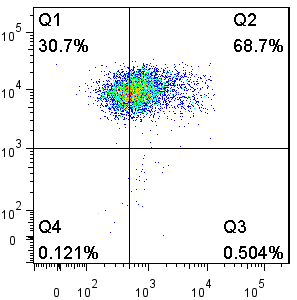
**

HepG2

**
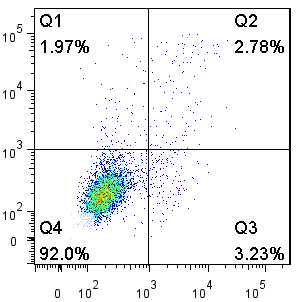
** **
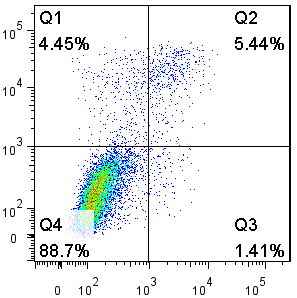
** **
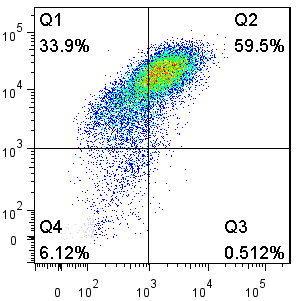
** **
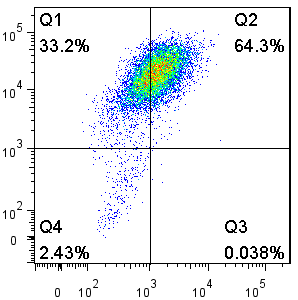
** **
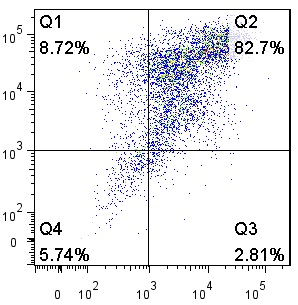
**

KB

PI

Annexin V FITC

Additional file 1: Figure S10: Flow cytometry analysis of cells (A549, HepG2, KB) after treatment with or without PTX/CUR/Au NRs@cRGD exposed to an 808 nm NIR laser at a 0.55 W cm^-2^ power density for 5 min.

**PTX/CUR/Au NRs@cRGD+laser**

**PTX/CUR/Au NRs@cRGD**

**Cur/Au NRs**

**KB**

**HepG2**

**A549**


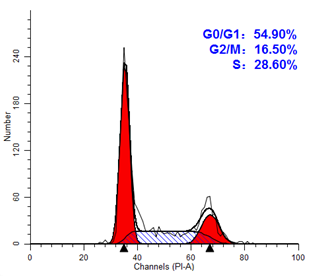

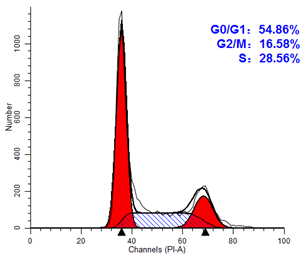

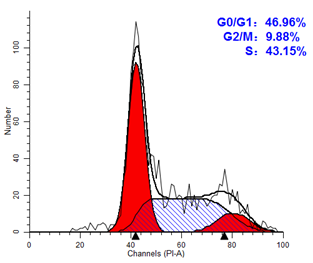


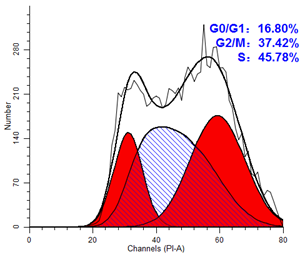

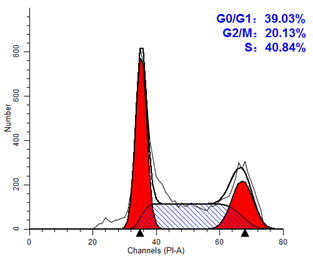

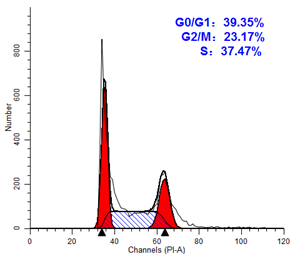


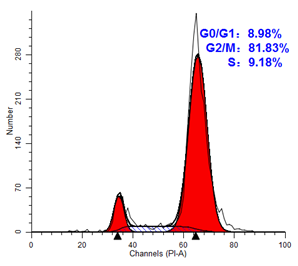

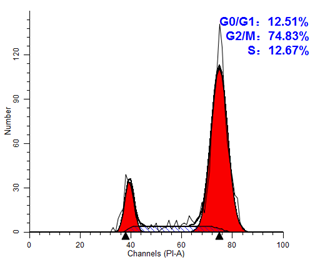

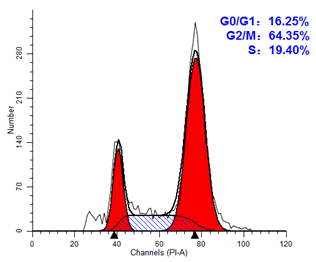


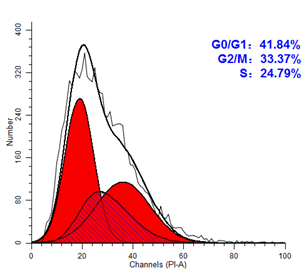

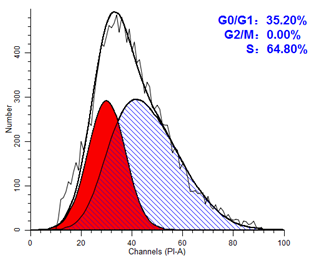

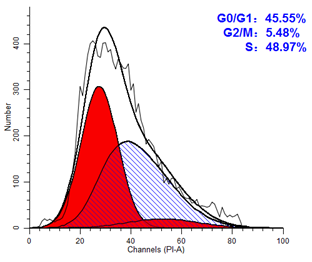


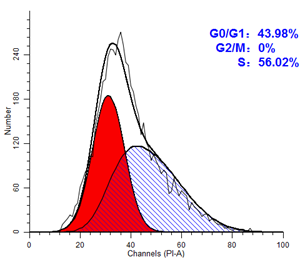

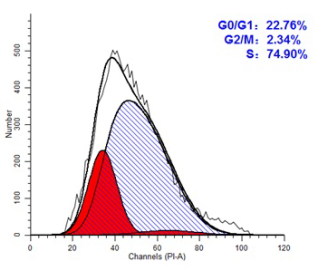

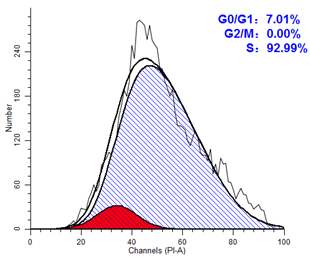


Additional file 1: Figure S11: Cell cycle analysis of A549, HepG2 and KB cells treated with PTX/CUR/Au NRs@cRGD with or without laser irradiation

**Control**

**PTX/Au NRs**

Additional file 1: Figure S12: Bio-distribution of nanoparticles (Au concentration % ID/g) in tumor tissues and organs at 6 h after the intravenous injection. Data is expressed as mean ± SD of the three experiments.

PTX/CUR/AuNRs@cRGD+laser

PEG/Au NRs@cRGD+laser

PTX/CUR/Au NRs@cRGD


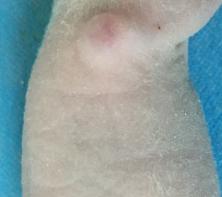


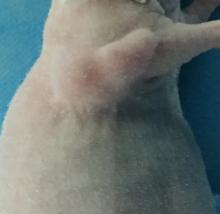

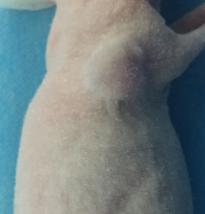

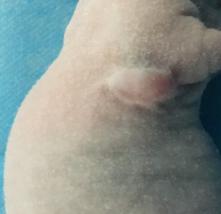

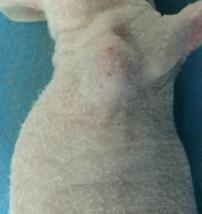

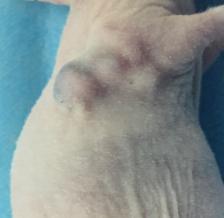


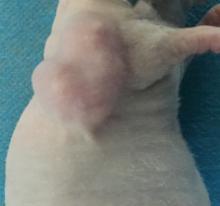

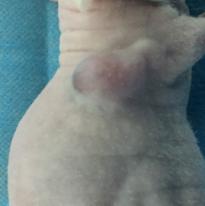

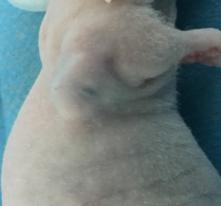

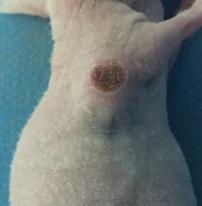

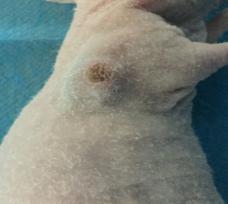


MUA-Cur+PTX

PBS

Pre

7 days

15 days

Additional file 1: Figure S13: Representative photographs of xenografted tumors taken before (day 0) and 7 and 15 days after treatment with PTX/CUR/Au NRs@cRGD at a power density of 0.95 W cm^-2^.

PTX/CUR/Au NRs@cRGD+ laser

PEG/Au NRs

@cRGD+ laser

PTX/CUR/

Au NRs@cRGD

MUA-Cur+PTX

PBS


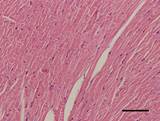

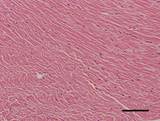

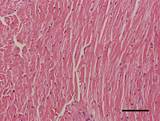

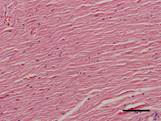

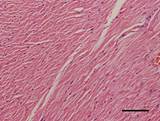


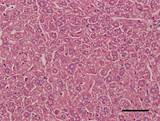

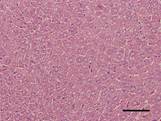

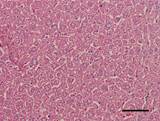

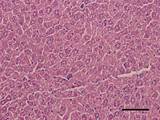

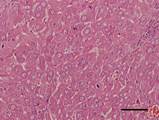


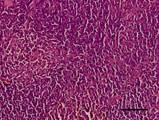

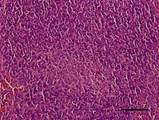

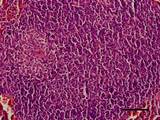

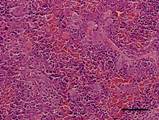

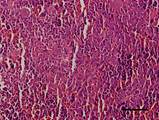


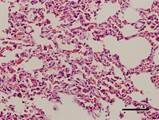

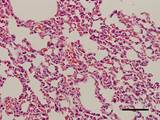

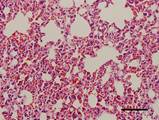

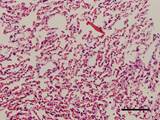

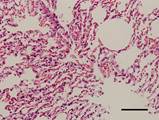


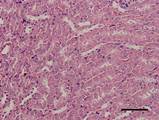

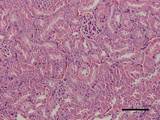

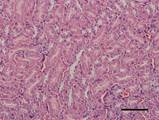

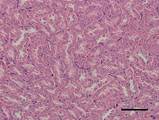

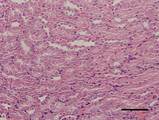


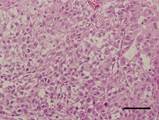

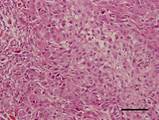

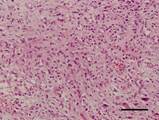

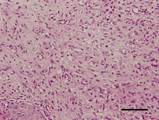

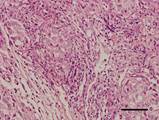


Heart

Liver

Spleen

Lung

Kidney

Tumor

Additional file 1: Figure S14: Representative H&E staining images of the tumor sections and major organs collected from different formulation-treated groups of mice on the 15th day after the PPTT therapy. Scale bar: 200 µm.

Additional file 1: Table S1: IC_50_ and CI Values of the Treatments in the A549, HepG2 and KB Cells

| Formulation | | IC_50_ (nM) | | CI |
| --- | --- | --- | --- | --- |
|  |  | PTX | CUR |  |
| A549 | Free PTX | 99.83 |  |  |
|  | Free CUR |  | 2336 |  |
|  | PTX/CUR/  Au NRs@cRGD | 60.3 | 120.6 | 0.6556 |
| HepG2 | Free PTX | 105.5 |  |  |
|  | Free CUR |  | 3894 |  |
|  | PTX/CUR/  Au NRs@cRGD | 57.74 | 115.46 | 0.5770 |
| KB | Free PTX | 69.08 |  |  |
|  | Free CUR |  | 334.9 |  |
|  | PTX/CUR/  Au NRs@cRGD | 26.78 | 53.56 | 0.5476 |

Additional file 1: Table S2: Calculated fraction of cell survival by additive interaction of PPTT and chemotherapy using PTX/CUR/Au NRs@cRGD

| Cell line PTX/CUR/Au NRs f_combination_ f_additive_  @cRGD (pM) |
| --- |
| A549 50 64.06 71.09  100 44.97 50.43  200 38.78 39.96  400 8.08 9.84  HepG2 50 59.28 76.05  100 43.97 46.75  200 23.69 25.80  400 10.56 11.56  KB 50 40.87 48.18  100 35.02 36.48  200 23.39 25.35  400 6.80 14.98 |

f_additive_ = f_PPTT_×f_chemotherapy_

Where f_additive_ is the fraction of surviving cells by additive interaction of PPTT and chemotherapy, f_PPTT_ is the fraction of surviving cells resulting from PPTT treatment, and f_chemotherapy_ is the fraction of surviving cells resulting from chemotherapy. f_combination_ is the fraction of surviving cells resulting from the PPTT + chemotherapy combination treatment . If f_combination_ is lower than f_additive_, there is a synergistic effect.
